# Association of Loneliness with Functional and Cognitive Status in Minor and Major Neurocognitive Disorders

**DOI:** 10.3390/life14101216

**Published:** 2024-09-24

**Authors:** Maria Claudia Moretti, Iris Bonfitto, Luciano Nieddu, Ivana Leccisotti, Savino Dimalta, Giovanni Moniello, Madia Lozupone, Antonello Bellomo, Francesco Panza, Carlo Avolio, Mario Altamura

**Affiliations:** 1 Department of Clinical and Experimental Medicine, University of Foggia, 71122 Foggia, Italy; mariaclaudiamoretti@gmail.com (M.C.M.); ivana.leccisotti@unifg.it (I.L.); antonello.bellomo@unifg.it (A.B.); mario.altamura@unifg.it (M.A.); 2 Department of Mental Health, Azienda Sanitaria Locale Foggia, 71121 Foggia, Italy; iris.84@hotmail.it (I.B.); dsmanfredonia@aslfg.it (S.D.); 3 Department of International Humanist and Social Studies, University of International Studies of Rome, 00147 Rome, Italy; l.nieddu@gmail.com; 4 Department of Medical Area, UOSD “Lungodegenza” P.O. di San Felice a Cancello, 81100 Caserta, Italy; giovanni.moniello@aslcaserta.it; 5 Department of Translational Biomedicine and Neuroscience “DiBraiN”, University of Bari Aldo Moro, 70124 Bari, Italy; 6 Department of Interdisciplinary Medicine, “Cesare Frugoni” Internal and Geriatric Medicine and Memory Unit, University of Bari Aldo Moro, 70124 Bari, Italy; f_panza@hotmail.com; 7 Department of Medical and Surgical Specialities, University of Foggia, 71122 Foggia, Italy; carlo.avolio@unifg.it

**Keywords:** loneliness, mild cognitive impairment, dementia, Alzheimer’s disease, MMSE, ADL, IADL, depression, multimorbidity

## Abstract

Background: Neurocognitive disorders (NCDs) have a variable decline in cognitive function, while loneliness was associated with cognitive impairment and increased dementia risk. In the present study, we examined the associations of loneliness with functional and cognitive status in patients with minor (mild cognitive impairment) and major NCDs (dementia). Methods: We diagnosed mild NCD (*n* = 42) and major NCD (*n* = 164) through DSM-5 criteria on 206 participants aged > 65 years using the UCLA 3-Item Loneliness Scale (UCLA-3) to evaluate loneliness, the activities of daily living (ADL) and the instrumental activities of daily living (IADL) scales to measure functional status, and Mini-Mental State Examination (MMSE) to assess cognitive functions. Results: In a multivariate regression model, the effect of loneliness on cognitive functions was negative in major (β = −1.05, *p* < 0.0001) and minor NCD (β = −0.06, *p* < 0.01). In the fully adjusted multivariate regression model (sex–age–education–multimorbidity–depressive symptoms–antidementia drug treatment), the effect of loneliness remained negative for major NCD and became positive for minor NCD (β = 0.09, *p* < 0.001). The effect of loneliness on IADL (β = −0.26, *p* < 0.0001) and ADL (β = −0.24, *p* < 0.001) showed a negative effect for major NCD across the different models, while for minor NCD, the effect was positive (IADL: β = 0.26, *p* < 0.0001; ADL: β = 0.05, *p* = 0.01). Minor NCD displayed different levels of MMSE (β = 6.68, *p* < 0.001) but not ADL or IADL, compared to major NCD for the same levels of loneliness. MANOVA pill test suggested a statistically significant and different interactive effect of loneliness on functional and cognitive variables between minor and major NCDs. Conclusions: We confirmed the relationships between loneliness and cognitive and functional status in major NCD, observing a novel trend in minor NCD.

## 1. Introduction

Dementia is a progressive neurological disorder affecting millions of individuals worldwide, posing significant challenges for both patients and their families [1]. It is characterised by a decline in cognitive functions, including memory and/or language loss, and changes in behaviour, which can profoundly disrupt the individual’s functional status [2], and may have a severe impact on global healthcare costs. Currently, there is talk of a “global aging phenomenon” due to the increase in life expectancy among the older age population and a rise in the prevalence of non-communicable diseases, such as dementia [3].

According to the World Health Organization, approximately 50 million people are currently living with dementia, with nearly 10 million new cases diagnosed every year. These numbers are projected to double every 20 years, highlighting the urgent need for global strategies to address this growing crisis [4], prompting intensified research efforts to better understand its causes and risk factors [5]. To date, 14 modifiable risk factors have been identified (among which social isolation), and it is estimated that about half of dementia cases could be prevented by modifying these factors [5].

Loneliness is considered a major public health issue. It is associated with accelerated physiological ageing and increased disability [6]. Loneliness had negative effects on mental health, including depression and sleep disturbances and was also associated with cognitive impairment and an increased risk of dementia [7,8,9]. Loneliness or emotional isolation is defined as a distressing feeling due to the difference between desired and perceived social connections [9]. Loneliness is a subjective and negative experience and the outcome of a cognitive evaluation of the mismatch between the quantity and quality of existing relationships and relationship standards.

Several studies have shown that in older adults experiencing loneliness, there was an association between loneliness and reduced cognitive functioning across multiple cognitive domains, as well as a heightened risk of dementia compared to non-lonely individuals [10,11]. It was shown that only chronic loneliness was associated with these negative outcomes, in contrast to transient loneliness, which might be considered protective against the development of dementia [12,13,14]. Loneliness may also be associated with poor health behaviours, such as decreased physical activity and more rapid motor decline in older age, as well as increased disability [15]. Furthermore, chronic loneliness, rather than transient loneliness, was an independent risk factor for functional disability in middle-aged and older adults [16]. However, findings were inconclusive [17]. The first objective of the present study was to examine the associations of loneliness with functional and cognitive status in patients with minor neurocognitive disorder (NCD) (mild cognitive impairment, MCI) and major NCD (dementia). The study also aimed to determine whether the impact of loneliness on functional status and cognition in different NCD groups can be explained by depressive symptoms, multimorbidity, lifestyle factors, and antidementia drug treatment.

## 2. Materials and Methods

This is a retrospective observational cohort study with data obtained by participants aged > 65 years afferent at the Center for Cognitive Disorders and Dementia (CDCD) in Manfredonia (Foggia, Apulia, South-East Italy) from January 2020 to April 2024. The collected data included demographic information such as age, gender, and education, with education expressed in years. The diagnosis of major NCD (dementia) and minor NCD (MCI) was made according to the Diagnostic and Statistical Manual of Mental Disorders, fifth edition (DSM-5) criteria [18].

Loneliness was measured using the adapted and Italian-validated University of California, Los Angeles (UCLA) Three-Item Loneliness Scale (UCLA-3), with responses categorised as “never/hardly ever”, “some of the time”, and “often”. The questions were scored from 1 to 3, and the scores were summed to produce a total score ranging from 3 to 9. The presence of loneliness was then dichotomised into “no loneliness” (scores 3 to 5) and “yes loneliness” (scores 6 to 9) [19].

Cognitive function was evaluated using the Mini-Mental State Examination (MMSE) [20]. The patient’s functional status was assessed using both the Instrumental Activities of Daily Living (IADL) [21] and Activities of Daily Living (ADL) scales [22], using the Italian versions. Higher scores indicate better preserved functional status. Multimorbidity status was defined as the presence of two or more chronic diseases, including the following conditions: cardiovascular-respiratory diseases, endocrine–metabolic diseases, gastric diseases, hepatic diseases, genitourinary diseases, and cancer [23]. Depressive symptoms were assessed using the 5-item Geriatric Depression Scale (GDS-5, score range 0–5 points) validated in Italian [24]. Each “yes” response was scored as 1, with total scores ranging from 0 to 5. A total score of 2 or higher was classified as indicative of depressive symptoms. Treatment with antidementia drugs was considered present when patients were receiving a specific medication with almost one of the following drugs: donepezil, rivastigmine, memantine, or galantamine.

### Statistical Analysis

Qualitative and quantitative variables are reported as frequencies and percentages and mean and standard deviation, respectively. Differences between minor NCD and major NCD groups for continuous variables were assessed using the *t*-test or Wilcoxon rank-sum test, as appropriate. For categorical variables, the chi-squared test or Fisher’s exact test was applied. To evaluate the impact of loneliness (UCLA-3) on the variability of cognitive functions (MMSE) and functional status (IADL and ADL), multivariate multiple regression models have been fitted to the data, allowing for interaction between loneliness and the group variables to assess possible differential effects between the two NCD groups (unadjusted model). Model 1 controlled for age, sex and education, model 2 additionally controlled multimorbidity, model 3 further controlled for depressive symptoms (GDS-5), and the final model (fully adjusted model) controlled for all the mentioned variables and antidementia drug treatment. Type II MANOVA Pillai’s trace was used to assess the significance of the effect of covariates on cognitive functions (MMSE) and functional status (IADL and ADL) jointly. Statistical significance was set at a *p*-value of < 0.05. The R v4.2.3 package was used to perform the analyses.

## 3. Results

The total sample of subjects with NCDs (minor and major NCD) consisted of 206 patients (55.8% women) with a mean age of 78.52 ± 7.08 years. The clinical and sociodemographic characteristics are described in Table 1. Out of the 206 patients enrolled in the analysis, 4 did not complete the UCLA-3 scale. Patients with major NCD amounted to 79.6% of the sample. Loneliness was present in 56.93% of the overall group. Comparisons of clinical and socio-demographic variables between minor and major NCDs were described in Table 2. The sample size, assuming a power of 0.80 and a significance level of 0.05, provides a two-sample *t* test able to detect an average effect size of 0.487 (the effect size for each test is listed in Table 2). Statistically significant differences were found for socio-demographic variables such as age and education (*p* < 0.0001), and a prevalence of females that can be considered marginally significant (*p* = 0.057) was also observed. As expected, statistically significant lower scores at MMSE (cognitive functions) and functional status investigated with IADLs and ADLs (*p* < 0.0001) were found in the dementia group (*p* < 0.0001). The percentage of lonely patients differed significantly among groups (86.09% in major NCD and 13.91% in minor NCD, respectively). There was no statistically significant difference in depressive symptoms between the two groups of NCDs (*p* = 0.24). All data are shown as mean ± standard deviation (SD), median for continuous variables and as *n* (%) for proportions. Significant values are labelled.

### 3.1. The Effect of Loneliness on Cognitive Functions

The effect of loneliness (UCLA-3) and both NCD groups on cognitive functions (MMSE) and functional status (IADL and ADL) have been reported in Table 3 and Table 4. In the unadjusted model, there was a significant negative effect of loneliness on MMSE both in the major NCD group (β = −1.05; *p* < 0.0001) and slightly in the minor NCD group (β = −0.06; *p* < 0.01). In the adjusted models, the effect of loneliness on MMSE for the major NCD group maintained the same trend, with a consistently significant negative interaction in model 1 after controlling for age, sex, and education (β = −0.92; *p* < 0.0001), in model 2 after controlling for multimorbidity (β = −0.94; *p* < 0.0001), in model 3 after controlling for depressive symptoms (β = −0.93; *p* < 0.0001), and in the fully adjusted model after controlling for antidementia drug treatment (β = −0.95; *p* < 0.0001). Concerning the minor NCD group, the effect of loneliness on MMSE changed significantly and became positive in the adjusted models. The interaction term in models 1 and 3 amounted to β = 0.14; *p* < 0.001. The association persisted in model 2 (β = 0.13; *p* < 0.001) and in the fully adjusted model (β = 0.09; *p* < 0.001). In the unadjusted model, the minor NCD group displayed higher (statistically different) levels of MMSE compared to the major NCD (β = 6.68; *p* < 0.001) at equal values of loneliness. This finding was confirmed in the adjusted models: model 1 (β = 5.06; *p* < 0.001), model 2 (β = 5.15; *p* < 0.001), model 3 (β = 5.14; *p* < 0.001), and in the fully adjusted (β = 6.38; *p* < 0.001).

### 3.2. The Effect of Loneliness on Instrumental Activities of Daily Living

In the unadjusted model, the effect of loneliness on IADLs showed a significant negative effect for the major NCD group (β = −0.42; *p* < 0.0001). This finding persisted in the adjusted models, respectively: model 2 (β = −0.41; *p* < 0.001), as well as in models 1 and 3 and the fully adjusted (β = −0.40; *p* < 0.001). The effect of loneliness on IADLs changed sign and became positive for the minor NCD group since from the unadjusted model (β = 0.23; *p* < 0.0001). The positive effect remained across the different models: model 2 (β = 0.25 and a *p*-value < 0.0001); models 1 and 3, and the fully adjusted model (β = 0.26 and a *p*-value < 0.0001). There were no significant differences in IADL scores for the same levels of loneliness between major and minor NCD groups (unadjusted model, *p* = 0.94; model 1, *p* = 0.72; models 2 and 3. *p* = 0.76; fully adjusted model, *p* = 0.77).

### 3.3. The Effect of Loneliness on Activities of Daily Living

In the unadjusted model, there was a significant negative effect of loneliness on ADL for the major NCD group (β = −0.28; *p* < 0.0001). This finding was confirmed in the adjusted models: Model 3 (β = −0.23 and a *p*-value < 0.0001), Models 1 and 2 and the fully adjusted model (β = −0.24; *p* < 0.001).

A significant positive effect of loneliness on ADL in the minor NCD group was found in the unadjusted model (β = 0.01; *p* = 0.02), in model 3 (β = 0.06; *p* = 0.01), and, respectively, in models 1 and 2 and the fully adjusted model (β = 0.05; *p* = 0.01). The group effect on ADL was not significant for the same levels of loneliness (unadjusted model, *p* = 0.13; model 1, *p* = 0.43; model 2, *p* = 0.44; model 3, *p* = 0.45; fully adjusted, *p* = 0.30).

### 3.4. Effect of Covariates on Cognitive Functions and Functional Status Jointly

Using Pillai’s Test, the effect of loneliness on cognitive functions (MMSE) and functional status (IADL and ADL) in both NCD groups was evaluated simultaneously. Results are presented in Table 4. The findings were significant both in the unadjusted model and after correcting for different covariates. Specifically, the Type II MANOVA suggested that a statistically significant effect of loneliness on the three dependent variables MMSE, IADL, and ADL was found (*p* < 0.0001), and this effect was significantly different across NCD groups (*p* < 0.001). Additionally, there was a statistically significant difference in the average values of the outcomes (MMSE, IADL, and ADL) between the two NCD groups (fully adjusted model: 0.31, *p* < 0.0001). Finally, considering the statistically significant interaction effect of loneliness on NCD groups, loneliness interacted with the three dependent variables (MMSE, IADLs, and ADLs) in a different way between minor and major NCD groups (fully adjusted model: 0.11, *p* < 0.0001).

## 4. Discussion

The present was a retrospective observational study involving 206 patients attending the CDCD in Manfredonia (Apulia, Italy). According to DSM-5 criteria, approximately 80% of the patients were diagnosed with major NCD (dementia) and about 20% with minor NCD (MCI). The overall prevalence of lonely older participants from Southern Italy amounted to 56.93% (respectively, 86.09% in major NCD and 13.91% in minor NCD). A negative association emerged between the level of loneliness and cognitive functions in patients with major NCD (about a 1-point reduction of MMSE for increasing values of UCLA-3) and a slightly positive correlation in patients with minor NCD (0.09-point increase in MMSE for increasing values of UCLA-3). Specifically, the negative interaction between loneliness and cognitive functions obtained in the crude model of the minor NCD group became positive after correcting for covariates (sex, education, age, multimorbidity, depressive symptoms, and antidementia drug treatment). Moreover, the minor NCD group displayed higher and statistically significant different levels of MMSE compared to the major NCD group (6.68 points) for the same values of loneliness. Furthermore, a slight negative correlation between loneliness and functional status (ADL and IADL, 0.24 and 0.40 points lower, respectively), was observed in patients with major NCD and a minimal positive correlation in patients with mild NCD. There were no significant differences in IADL and ADL scores at the same levels of loneliness between mild and major NCD groups. Finally, an interactive effect of loneliness was confirmed on the variables considered (cognitive and functional status), with statistically significant differences between the two NCD groups.

While the present findings confirmed the existing literature concerning the relationship between loneliness and cognition for the major NCD group, our results added something different about the minor NCD group. The present findings were in line with previous studies showing a detrimental effect of loneliness on subsequent cognitive decline or dementia [17]. According to the WHO, dementia is currently the seventh leading cause of death and one of the main causes of disability and dependence among the older age population worldwide [4]. Dementia prevention and early diagnosis should be considered a public health priority, not only due to the increasing number of cases but also because of the severe functional limitations it entails [25]. Additionally, dementia requires significant long-term care and substantial financial and social costs. It is therefore important to identify and counteract factors that may contribute to cognitive decline, such as loneliness, which is closely associated with dementia in a bidirectional way. According to a biopsychosocial model, loneliness may biologically prepare one for increased threats or stressors by eliciting the fight-or-flight response, which could cause dysregulation in multiple physiological systems. These physiological dysregulations included heightened low-grade systemic inflammation, renal injury, and deregulated metabolic health [26]. Furthermore, loneliness is a risk factor for neuropsychiatric symptoms, especially for depressive symptoms and psychosis, including delusions and hallucinations [27].

Loneliness was considered a public health issue [7], and the influence of loneliness on the risk for dementia is comparable in size with other well-established risk factors for cognitive decline and dementia [5]. The present study, in fact, highlighted how patients affected by dementia may experience stronger feelings of loneliness for lower levels of cognitive impairment. Loneliness can contribute to the onset or to the worsening of dementia, as the lack of social and intellectual stimulation may accelerate cognitive decline. On the other hand, the progression of dementia can lead to increased loneliness, as patients may become more withdrawn and less able to participate in conversations and social activities. This was consistent with other recent studies on the same topic [26,27]. Indeed, Freak-Poli and colleagues [28] conducted a study based on data from two longitudinal studies that highlighted a correlation between loneliness and cognitive decline measured by the MMSE, as well as an increased risk of dementia. Furthermore, loneliness (both persistent/chronic and transient/situational) was associated with an increased risk of all-cause dementia, especially if loneliness was experienced before the age of 70 years [29]. On the other hand, Griffin and colleagues [30] confirmed the association of loneliness with cognitive decline but did not find evidence of a more rapid decline at follow-up.

Conversely, limited evidence suggested a potential effect of loneliness on the minor NCD group (MCI). In addition, some recent cross-sectional studies exploring this association have reported conflicting results [31,32]. Although our study highlighted that in the minor NCD group, as the level of loneliness increased, cognitive performance measured by MMSE decreased in the unadjusted model, this finding was inverted in the adjusted models. This finding underlined the uncertain relationship between MCI and the neuropsychological characteristics of the construct of loneliness and the value given by age, sex, education, multimorbidity, depressive symptoms, and antidementia drug treatment to cognition in the minor NCD group. Although the complexity of loneliness (with its subdivision into emotional loneliness and social loneliness), the UCLA-3 was considered an instrument based on a multidimensional perspective. We agreed that the dichotomisation of loneliness scores oversimplified the complexity of loneliness despite its efficacious psychometric properties [19,33]. We could suppose that in the initial and transient phase of MCI, the higher level of cognition makes aware participants with MCI of the experience of loneliness. This could be the explanation for why individuals with MCI with higher cognitive levels perceived themselves as lonelier. Although this association was weak, it is intriguing, as it may reflect that some individuals with MCI could respond to loneliness by attempting to maintain or stimulate their cognitive abilities. Other studies appeared to be sparse on this topic, although this finding was also supported by Yu and colleagues [34,35], who observed that individuals with MCI, being aware of their initial cognitive impairment, would experience greater feelings of loneliness. Finally, living arrangements could be a modifier of the associations of loneliness with adverse health-related outcomes in community-dwelling older adults, as recently found by the Chinese Longitudinal Healthy Longevity Survey [36]. Unfortunately, we did not have this data in our clinical population.

The present study also examined the effect of loneliness on functional status, as measured by the IADL and ADL scales. We found that loneliness was associated with poorer functioning in individuals with major NCD (dementia), whereas in individuals with minor NCD (MCI), it was associated with better functional status. Therefore, patients with dementia who felt lonely tended to lose their ability to perform daily activities (both basic and complex) more rapidly. This could be because loneliness may exacerbate the symptoms of dementia, such as confusion, memory loss, and the inability to care for oneself. Chronic loneliness, rather than transient loneliness, was an independent risk factor for functional disability as measured with ADL/IADL in middle-aged and older adults, especially for women [16]. According to Shankar and colleagues [37], increased loneliness may be associated with a higher likelihood of physical inactivity and multiple health-risk behaviours. In contrast, in individuals with minor NCD (MCI), according to the present study and according to the same trend with MMSE scores, loneliness might encourage some people to be more independent or to find ways to maintain their functional abilities. This could also reflect a phenomenon where patients with MCI who perceive loneliness attempt to be more active and strive to maintain their functioning to counteract feelings of isolation.

This finding was not supported by the existing literature. Conversely, Guo and colleagues [38] did not find a significant association between loneliness and ADL/IADL functional disability. Finally, the complex analysis through MANOVA Pillai’s Test suggested that in both NCD groups existed a statistically significant and different interactive effect of loneliness on the three dependent variables considered (cognitive and functional status). Our study was the first to evaluate the interactive effect of loneliness on functional and cognitive status, finding a statistically significant and different (maybe inversed) effect between major NCD-dementia and minor NCD-MCI groups.

We must acknowledge some limitations of the present study. First, this study was preliminary and conducted with a relatively small number of participants but appropriate to test the specific aim of the study. Moreover, given the clinical nature of the sample examined, the results cannot be generalised to the whole population of older people. Distal demographic factors (age, gender, race/ethnicity) operate through structural factors (income, education) and, in turn, through health, social roles, and stress—proximal factors that are more directly associated with social network size and relationship quality [39,40]. Finally, this study could not identify all the relevant factors which may be associated with loneliness, depression, or cognitive function, such as socioeconomic status, social support networks, or previous mental health history.

## 5. Conclusions

In conclusion, loneliness appeared to have a negative effect on individuals already in an advanced stage of cognitive impairment (major NCD-dementia), while it may paradoxically have a “protective” or stimulating effect in the early stages of cognitive decline (minor NCD-MCI). However, it is important to interpret these findings with caution and consider them in the context of further research. Without further analysis, we cannot exclude both a deeper causal relationship and some artefacts of the methodological approach (i.e., cross-sectional analysis). Further research is needed about the influence of other covariates (i.e., social network) or longitudinal analysis with a larger sample of minor NCDs or for different cut-offs of cognitive and functional status.

## Figures and Tables

**Table 1:** Sociodemographic and clinical characteristics of the whole sample of older subjects with neurocognitive disorders (NCDs) (*N* = 206).

	Mean ± SD or *n*	Median or %
Major NCD	164	79.6
Minor NCD	42	20.4
Age	78.52 ± 7.08	79
Education	6.22 ± 3.82	5
Females	115	55.8
Males	91	44.2
UCLA-3	5.20 ± 2.58	6
Loneliness		
Yes	115	56.93
No	87	43.07
MMSE	17.81 ± 8.14	18
ADL	4.02 ± 2.10	5
IADL	3.60 ± 2.82	3
Multimorbidity		
Yes	106	51.5
No	100	48.5
GDS-5	1.91 ± 1.64	2
Antidementia drug treatment		
Yes	127	61.7
No	79	38.3

UCLA-3, University of California, Los Angeles (UCLA) Three-Item Loneliness Scale; MMSE, Mini Mental State Examination; ADL, Index of Independence in Activities of Daily Living; IADL, Instrumental Activities of Daily Living Scale; GDS-5, 5-item Geriatric Depression Scale; Multimorbidy score: defined as the presence of two or more chronic diseases among the following pathologies: stroke, hypertension, chronic obstructive pulmonary disease, asthma and diabetes mellitus.

**Table 2:** Sociodemographic and clinical characteristics of the whole sample according to minor and major neurocognitive disorders (NCDs) groups.

	Major NCD (*n* = 164)		Minor NCD (*n* = 42)			
	Mean ± SD or *n*	Median or %	Mean ± SD or *n*	Median or %	*p*-Value	Effect Size
Proportions (%)	164	79.6	42	20.4		
Age	79.73 ± 6.61	80.0	73.81 ± 6.98	74.5	***p* < 0.0001**	0.487
Education	5.50 ± 3.38	5.0	9.02 ± 4.19	8.0	***p* < 0.0001**	0.487
Females	97	84.35	18	16.65	*p* = 0.06	0.195
Males	67	73.63	24	26.37		
UCLA-3	5.58 ± 2.40	6.0	3.76 ± 2.76	4.0	***p* < 0.0001**	0.488
Loneliness (%)	99	86.09	16	13.91	***p* < 0.001**	0.197
MMSE	15.22 ± 7.07	16.0	27.93 ± 1.05	28.0	***p* < 0.0001**	0.487
ADL	3.52 ± 2.08	4.0	5.98 ± 0.15	6.0	***p* < 0.0001**	0.487
IADL	2.94 ± 2.66	2.5	6.19 ± 1.76	5.0	***p* < 0.0001**	0.487
Multimorbidity						
Yes	82	77.36	24	22.64	*p* = 0.41	0.195
No	82	82.0	18	18.0		
GDS-5	1.98 ± 1.68	2.00	1.67 ± 1.49	1.5	*p* = 0.25	0.487
Antidementia drug treatment						
Yes	122	96.06	5	3.94	***p* < 0.0001**	0.195
No	42	53.16	37	46.84		

UCLA-3, University of California, Los Angeles (UCLA) Three-Item Loneliness Scale; MMSE, Mini Mental State Examination; ADL, Index of Independence in Activities of Daily Living; IADL, Instrumental Activities of Daily Living Scale; GDS-5, 5-item Geriatric Depression Scale. Significant values are bold.

**Table 3:** Multivariate regression models on the effect of loneliness for minor and major neurocognitive disorders (NCDs) groups on cognitive functions (Mini-Mental State Examination, MMSE) and functional status (Activities of Daily Living, ADL and Instrumental Activities of Daily Living Scale, IADL).

	Unadjusted Model		Model 1		Model 2		Model 3		Fully Adjusted Model	
MMSE	β	*p*-Value	β	*p*-Value	β	*p*-Value	β	*p*-Value	β	*p*-Value
UCLA-3-Major NCD	−1.05	***p* < 0.0001**	−0.92	***p* < 0.0001**	−0.94	***p* < 0.0001**	0.93	***p* < 0.0001**	−0.95	***p* < 0.0001**
Major NCD-Minor NCD	6.68	***p* < 0.001**	5.06	***p* < 0.001**	5.15	***p* < 0.0001**	5.14	***p* < 0.001**	6.38	***p* < 0.001**
UCLA-3-Minor NCD	−0.06	***p* < 0.01**	0.14	***p* < 0.001**	0.13	***p* < 0.001**	0.14	***p* < 0.001**	0.09	***p* < 0.001**
Sex			0.62	*p* = 0.44	−0.62	*p* = 0.44	−0.63	*p* = 0.43	−0.54	*p* = 0.50
Age			−0.15	***p* = 0.01**	−0.14	***p* = 0.02**	−0.15	***p* = 0.02**	−0.14	***p* = 0.03**
Education			0.23	***p* < 0.05**	0.23	***p* = 0.05**	0.23	***p* = 0.04**	0.22	*p* = 0.06
Multimorbidity					−0.62	*p* = 0.43	−0.60	*p* = 0.44	−0.72	*p* = 0.36
GDS-5							−0.05	*p* = 0.84	−0.01	*p* = 0.96
Antidementia drug treatment									1.77	*p* = 0.06
**IADL**	**β**	***p*** **-Value**	**β**	***p*** **-Value**	**β**	***p*** **-Value**	**β**	***p*** **-Value**	**β**	***p*** **-Value**
UCLA-3-Major NCD	−0.42	***p* < 0.0001**	−0.40	***p* < 0.0001**	−0.41	***p* < 0.0001**	−0.40	***p* < 0.0001**	−0.40	***p* < 0.0001**
Major NCD-Minor NCD	−0.06	*p* = 0.94	−0.27	*p* = 0.72	−0.23	*p* = 0.76	−0.23	***p* < 0.0001**	−0.23	*p* = 0.78
UCLA-3-Minor NCD	0.23	***p* < 0.0001**	0.26	***p* < 0.0001**	0.25	***p* < 0.0001**	0.26	***p* < 0.0001**	0.26	***p* < 0.0001**
Sex			−1.47	***p* < 0.0001**	−1.47	***p* < 0.0001**	−1.48	***p* < 0.0001**	−1.48	***p* < 0.0001**
Age			−0.07	***p* < 0.001**	−0.06	***p* < 0.001**	−0.07	***p* < 0.001**	−0.07	***p* < 0.001**
Education			0.03	*p* = 0.56	0.02	*p* = 0.59	0.02	*p* = 0.60	0.02	*p* = 0.60
Multimorbidity					−0.29	*p* = 0.34	−0.29	*p* = 0.35	−0.29	*p* = 0.36
GDS-5							−0.02	*p* = 0.81	−0.02	*p* = 0.81
Antidementia drug treatment									0.01	***p* < 0.0001**
**ADL**	**β**	***p*** **-Value**	**β**	***p*** **-Value**	**β**	***p*** **-Value**	**β**	***p*** **-Value**	**β**	***p*** **-Value**
UCLA-3-Major NCD	−0.28	***p* < 0.0001**	−0.24	***p* < 0.0001**	−0.24	***p* < 0.0001**	−0.23	***p* < 0.0001**	−0.24	***p* < 0.0001**
Major NCD-Minor NCD	0.87	*p* = 0.13	0.46	*p* = 0.43	0.45	*p* = 0.44	0.44	*p* = 0.45	0.65	*p* = 0.30
UCLA-3-Minor NCD	0.01	***p* = 0.02**	0.05	***p* = 0.01**	0.05	***p* = 0.01**	0.06	***p* = 0.01**	0.05	***p* = 0.01**
Sex			−0.05	*p* = 0.82	−0.05	*p* = 0.83	−0.07	*p* = 0.78	−0.05	*p* = 0.83
Age			−0.05	***p* = 0.01**	−0.05	***p* = 0.01**	−0.05	***p* < 0.001**	−0.05	***p* = 0.01**
Education			0.03	*p* = 0.36	0.03	*p* = 0.36	0.03	*p* = 0.38	0.03	*p* = 0.40
Multimorbidity					0.02	*p* = 0.93	0.03	*p* = 0.89	0.02	*p* = 0.95
GDS-5							−0.05	*p* = 0.48	−0.05	*p* = 0.54
Antidementia drug treatment									0.29	*p* = 0.33

UCLA-3, University of California, Los Angeles (UCLA) Three-Item Loneliness Scale; GDS, 5-item Geriatric Depression Scale. Significant values are bold.

**Table 4:** Type II MANOVA Pillai’s test analysing the interactive effect of loneliness on the three dependent variables evaluating cognitive functions (Mini-Mental State Examination, MMSE) and functional status (Activities of Daily Living, ADL and Instrumental).

	Unadjusted Model		Model 1		Model 2		Model 3		Fully Adjusted Model	
	Pillai’s Test	*p*-Value	Pillai’s Test	*p*-Value	Pillai’s Test	*p*-Value	Pillai’s Test	*p*-Value	Pillai’s Test	*p*-Value
UCLA-3-Major NCD	0.12	***p* < 0.0001**	0.09	***p* < 0.0001**	0.09	***p* < 0.0001**	0.09	***p* < 0.0001**	0.09	***p* < 0.0001**
Major NCD-Minor NCD	0.38	***p* < 0.0001**	0.32	***p* < 0.0001**	0.33	***p* < 0.0001**	0.33	***p* < 0.0001**	0.31	***p* < 0.0001**
UCLA-3-Minor NCD	0.09	***p* < 0.001**	0.11	***p* < 0.0001**	0.11	***p* < 0.0001**	0.11	***p* < 0.0001**	0.11	***p* < 0.0001**
Sex			0.18	***p* < 0.0001**	0.19	***p* < 0.0001**	0.19	***p* < 0.0001**	0.19	***p* < 0.0001**
Age			0.04	***p* = 0.04**	0.04	***p* = 0.05**	0.04	***p* = 0.05**	0.04	*p* = 0.06
Education			0.03	*p* = 0.17	0.02	*p* = 0.18	0.02	*p* = 0.18	0.02	*p* = 0.20
Multimorbidity					0.02	*p* = 0.33	0.02	*p* = 0.31	0.02	*p* = 0.30
GDS-5							0.00	*p* = 0.84	0.00	*p* = 0.84
Antidementia drug treatment									0.03	*p* = 0.14

UCLA-3, University of California, Los Angeles (UCLA) Three-Item Loneliness Scale; GDS, 5-item Geriatric Depression Scale. Significant values are bold.

## Data Availability

The raw data supporting the conclusions of this article will be made available by the authors on request.

## References

[1] Lucca U., Tettamanti M., Tiraboschi P., Logroscino G., Landi C., Sacco L., Garrì M., Ammesso S., Biotti A., Gargantini E. (2020). Incidence of dementia in the oldest-old and its relationship with age: The Monzino 80-plus population-based study. Alzheimer’s Dement..

[2] Knopman D.S., Amieva H., Petersen R.C., Chételat G., Holtzman D.M., Hyman B.T., Nixon R.A., Jones D.T. (2021). Alzheimer disease. Nat. Rev. Dis. Primers.

[3] Guarnera J., Yuen E., Macpherson H. (2023). The Impact of Loneliness and Social Isolation on Cognitive Aging: A Narrative Review. J. Alzheimers Dis. Rep..

[4] World Health Organization Dementia. Last Updated: 15 August 2023. https://www.who.int/news-room/fact-sheets/detail/dementia.

[5] Livingston G., Huntley J., Liu K.Y., Costafreda S.G., Selbæk G., Alladi S., Ames D., Banerjee S., Burns A., Brayne C. (2024). Dementia prevention, intervention, and care: 2024 report of the Lancet standing Commission. Lancet.

[6] Hawkley L.C., Cacioppo J.T. (2010). Loneliness matters: A theoretical and empirical review of consequences and mechanisms. Ann. Behav. Med..

[7] House J.S., Landis K.R., Umberson D. (1988). Social relationships and health. Science.

[8] Surkalim D.L., Luo M., Eres R., Gebel K., van Buskirk J., Bauman A., Ding D. (2022). The prevalence of loneliness across 113 countries: Systematic review and meta-analysis. BMJ.

[9] Cohen-Mansfield J., Hazan H., Lerman Y., Shalom V. (2016). Correlates and predictors of loneliness in older-adults: A review of quantitative results informed by qualitative insights. Int. Psychogeriatr..

[10] Akhter-Khan S.C., Tao Q., Ang T.F.A., Itchapurapu I.S., Alosco M.L., Mez J., Piers R.J., Steffens D.C., Au R., Qiu W.Q. (2021). Associations of loneliness with risk of Alzheimer’s disease dementia in the Framingham Heart Study. Alzheimer’s Dement..

[11] Solfrizzi V., Scafato E., Custodero C., Piazzolla G., Capogna L., Procaccio A., Gandin C., Galluzzo L., Ghirini S., Matone A. (2023). IPREA Working Group. Biopsychosocial frailty and mild cognitive impairment subtypes: Findings from the Italian project on the epidemiology of Alzheimer’s disease (IPREA). Alzheimer’s Dement..

[12] Victor C.R. (2020). Is Loneliness a Cause or Consequence of Dementia? A Public Health Analysis of the Literature. Front. Psychol..

[13] Salinas J., Beiser A.S., Samra J.K., O’Donnell A., DeCarli C.S., Gonzales M.M., Aparicio H.J., Seshadri S. (2022). Association of Loneliness with 10-Year Dementia Risk and Early Markers of Vulnerability for Neurocognitive Decline. Neurology.

[14] Sutin A.R., Stephan Y., Luchetti M., Terracciano A. (2020). Loneliness and Risk of Dementia. J. Gerontol. B Psychol. Sci. Soc. Sci..

[15] Emerson E., Stancliffe R.J., Aitken Z., Bailie J., Bishop G.M., Badland H., Llewellyn G., Kavanagh A.M. (2023). Disability and loneliness in the United Kingdom: Cross-sectional and longitudinal analyses of trends and transitions. BMC Public Health.

[16] Qi X., Belsky D.W., Yang Y.C., Wu B. (2023). Association between Types of Loneliness and Risks of Functional Disability in Older Men and Women: A Prospective Analysis. Am. J. Geriatr. Psychiatry.

[17] Lara E., Martín-María N., De la Torre-Luque A., Koyanagi A., Vancampfort D., Izquierdo A., Miret M. (2019). Does loneliness contribute to mild cognitive impairment and dementia? A systematic review and meta-analysis of longitudinal studies. Ageing Res. Rev..

[18] American Psychiatric Association (2022). Diagnostic and Statistical Manual of Mental Disorders.

[19] Boffo M., Mannarini S., Munari C. (2012). Exploratory structure equation modeling of the UCLA Loneliness Scale: A contribution to the Italian adaptation. TPM-Test. Psychom. Methodol. Appl. Psychol..

[20] Magni E., Binetti G., Bianchetti A., Rozzini R., Trabucchi M. (1996). Mini-Mental State Examination: A normative study in Italian elderly population. Eur. J. Neurol..

[21] Quesada J.J., Ferrucci L., Calvani D., Valente C., Salani B., Bavazzano A. (1997). Formal education as an effect modifier of the relationship between Mini-Mental State Examination score and IADLs disability in the older population. Aging.

[22] Pezzuti L., Artistico D., Mallozzi P., Sellitto M., Vozella M. (2009). Validazione del punteggio scalare per la scala adl (attività di vita quotidiana). Ric. Psicol..

[23] World Health Organization Multimorbidity. https://apps.who.int/iris/handle/10665/252275.

[24] Galeoto G., Sansoni J., Scuccimarri M., Bruni V., De Santis R., Colucci M., Valente D., Tofani M. (2018). A Psychometric Properties Evaluation of the Italian Version of the Geriatric Depression Scale. Depress. Res. Treat..

[25] Tomida K., Shimoda T., Nakajima C., Kawakami A., Shimada H. (2024). Social Isolation/loneliness and Mobility Disability among Older Adults. Curr. Geriatr. Rep..

[26] Yu K., Siang Ng T.K. (2023). Investigating Biological Pathways Underpinning the Longitudinal Association between Loneliness and Cognitive Impairment. J. Gerontol. A Biol. Sci. Med. Sci..

[27] Sun W., Matsuoka T., Oba H., Narumoto J. (2021). Importance of loneliness in behavioral and psychological symptoms of dementia. Int. J. Geriatr. Psychiatry.

[28] DiNapoli E.A., Wu B., Scogin F. (2014). Social isolation and cognitive function in Appalachian older adults. Res. Aging.

[29] Shankar A., Hamer M., McMunn A., Steptoe A. (2013). Social isolation and loneliness: Relationships with cognitive function during 4 years of follow-up in the English Longitudinal Study of Ageing. Psychosom. Med..

[30] Freak-Poli R., Wagemaker N., Wang R., Lysen T.S., Ikram M.A., Vernooij M.W., Dintica C.S., Vernooij-Dassen M., Melis R.J.F., Laukka E.J. (2022). Loneliness, Not Social Support, Is Associated with Cognitive Decline and Dementia across Two Longitudinal Population-Based Cohorts. J. Alzheimer’s Dis..

[31] Khaing K., Dolja-Gore X., Nair B.R., Byles J., Attia J. (2024). The Effect of Temporal Persistence of Loneliness on Dementia: A Longitudinal Analysis From the Hunter Community Study. Int. J. Geriatr. Psychiatry.

[32] Griffin S.C., Mezuk B., Williams A.B., Perrin P.B., Rybarczyk B.D. (2020). Isolation, Not Loneliness or Cynical Hostility, Predicts Cognitive Decline in Older Americans. J. Aging Health.

[33] Yanguas J., Pinazo-Henandis S., Tarazona-Santabalbina F.J. (2018). The complexity of loneliness. Acta Biomed..

[34] Yu J., Lam C.L.M., Lee T.M.C. (2016). Perceived loneliness among older adults with mild cognitive impairment. Int. Psychogeriatr..

[35] Kwon D.-Y., Jung J.-M., Park M.H. (2017). Loneliness in elderly patients with mild cognitive impairment: A pilot study. Dement. Neurocogn. Disord..

[36] Wei K., Liu Y., Yang J., Gu N., Cao X., Zhao X., Jiang L., Li C. (2022). Living arrangement modifies the associations of loneliness with adverse health outcomes in older adults: Evidence from the CLHLS. BMC Geriatr..

[37] Shankar A., McMunn A., Banks J., Steptoe A. (2011). Loneliness, social isolation, and behavioral and biological health indicators in older adults. Health Psychol..

[38] Guo L., An L., Luo F., Yu B. (2020). Social isolation, loneliness and functional disability in Chinese older women and men: A longitudinal study. Age Ageing.

[39] Hawkley L.C., Hughes M.E., Waite L.J., Masi C.M., Thisted R.A., Cacioppo J.T. (2008). From social structural factors to perceptions of relationship quality and loneliness: The Chicago Health, Aging, and Social Relations Study. J. Gerontol. Soc. Sci..

[40] Barjaková M., Garnero A., d’Hombres B. (2023). Risk factors for loneliness: A literature review. Soc. Sci. Med..

